# Complete mitochondrial genome sequence of *Glyptothorax annandalei* in the Yarlung Zangbo River, Tibet

**DOI:** 10.1080/23802359.2021.1911701

**Published:** 2021-04-15

**Authors:** Xiaowan Lin, Lei Li, Hongyu Jin, Xing Jin, Bo Ma

**Affiliations:** a College of Life Science and Technology, Harbin Normal University, Harbin, P. R. China; b Heilongjiang River Fisheries Research Institute, Chinese Academy of Fishery Sciences, Harbin, P. R. China

**Keywords:** *Glyptothorax annandalei*, mitochondrial genome, complete genome

## Abstract

*Glyptothorax annandalei* belongs to the family of Sisoridae, Glyptothorax. It is distributed in the Yarlung Zangbo River of southwestern China. In this study, we first published the complete mitochondrial genome sequence of *Glyptothorax annandalei*, which was 16,541 bp in length. This genome consists of two rRNA genes, 22 tRNA genes, 13 protein-coding genes (PCGs), and a putative control region. The overall base composition was for A(31.25%,) for T(25.67%), for C(27.66%), for G(15.42%). The PCGs start with a traditional ATG except for COX1 start with GTG, respectively, and end with stop codon TAA, TAG, or a single T base. All tRNA have the typical clover-leaf structure. The phylogenetic tree of the whole mitogenome sequence is constructed by maximum likelihood (ML) method and the phylogenetic relationship among the family Sisoridae is further analyzed. We expect to provide the theoretical basis for the further study of the phylogenetic relationship, taxonomic status, conservation and management of genetic resources of Sisoridae catfishes.

## Introduction

*Glyptothorax annandalei* (Hora 1938) belongs to Sisoridae, Glyptothorax, and is distributed in the Yarlung Zangbo River . In this study, *G. annandaleiwere* collected from the Motuo section (29.18_N, 94.16_E) and were stored in the fish specimen room of Heilongjiang River Fisheries Research Institute, Chinese Academy of Fishery Sciences (specimen Accession number: XZ-5-001). We used the phenol-chloroform method to extract whole genome of *G. annandaleiand* (Taggart et al. 1992). We first published the complete mitochondrial genome of *G. annandalei and* then analyzed the whole mitochondrial genome sequence.

The complete mitochondrial genone of *G. annandalei* and was 16,541 bp and has been deposited in GenBank (accession number: MN396887). The overall base composition of *G. annandalei* was for A(31.25%,) for T(25.67%), for C(27.66%), for G(15.42%). And consisting of two rRNA genes, 22 tRNA genes, 13 protein-coding genes (PCGs), and a putative control region. There are two kinds of start codons for the protein genes of *G. annandalei*, excepting that the start codon of COX1 is GTG, the other genes are all ATG; there are three kinds of stop codons (TAA, TAG, T––), among these codons NAD1, COX1, ATP8, NAD4L, NAD5, and NAD6 is terminated by TAA, NAD2, NAD3, and COX3 are terminated by TAG, and COX2, ATP6, NAD4 and Cyt *b* are terminated by an incomplete codon T––. The tRNA genes encoded by the L chain are tRNA-Gln, tRNA-Ala, tRNA-Asn, tRNA-Cys, tRNA-Tyr, tRNA-Ser^UCN^, tRNA-Glu and tRNA-Pro, and the remaining genes are encoded by the H chain. The secondary structure prediction diagram showed that all the 22 tRNA folded were typical clover structures.

**Figure 1.**
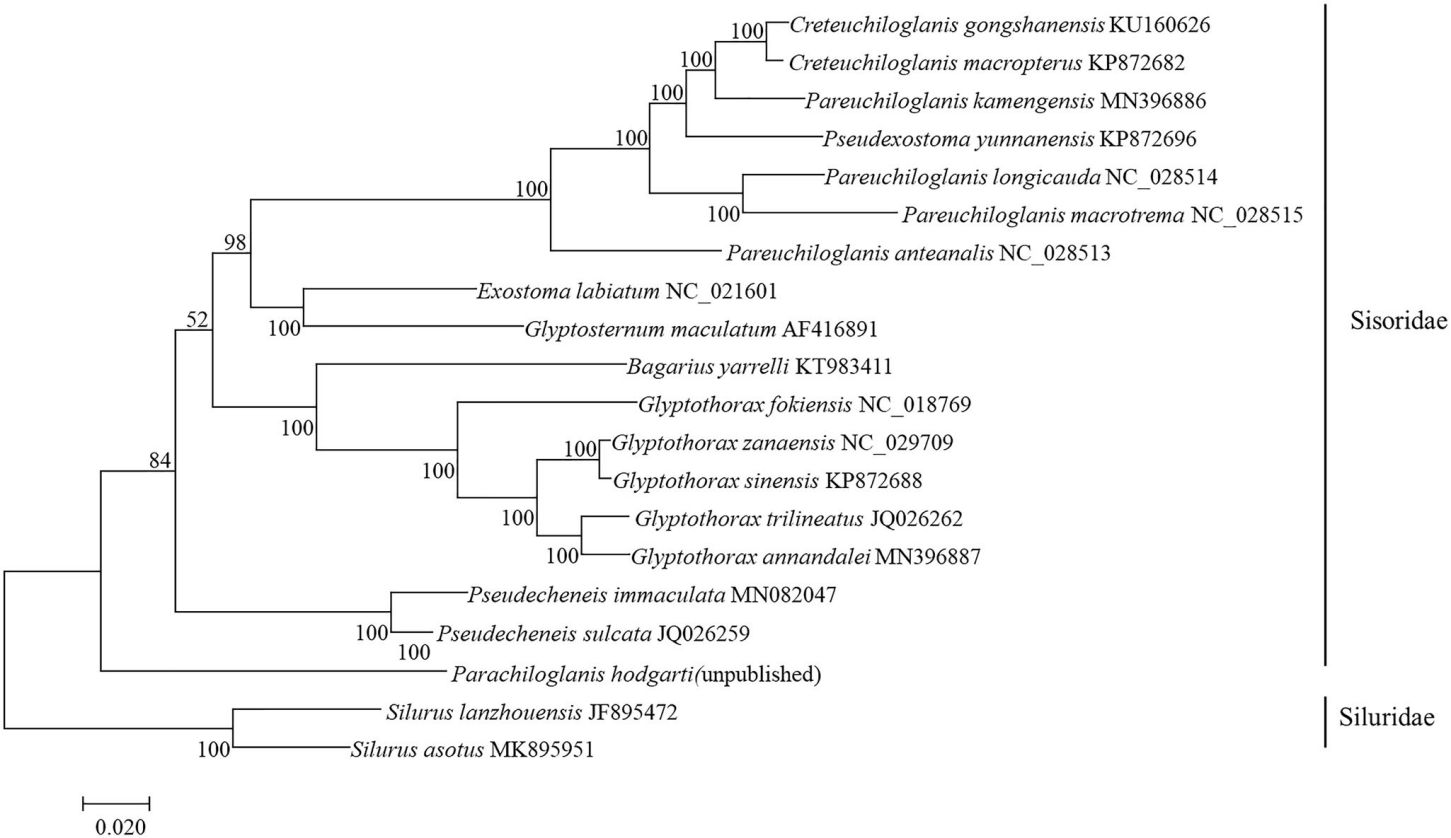
The phylogenetic tree (maximum likelihood) of *Glyptothorax annandalei* in this study and other 19 species (*Silurus lanzhouensis*, *Silurus asotus* belong to the Siluridae, and were used as outgroups of the Siluriidae. The rest are Sisoridae) based on the whole mitogenome sequence. Shown next to the nodes are bootstrap support values based on 1000 replicates.

In this study, 15 species are used to build the phylogenetic tree of the whole mitogenome sequence by using the maximum likelihood (ML) method (Figure 1). The monosyllabicity of Sisoridae catfishes is supported. The *G. annandalei* in this study and the other three selected Glyptothorax fishes also form a monophyletic group. In addition, Glyptothorax fishes have always been regarded as a group with an uncertain phylogenetic position in the study of Sisoridae catfishes. The results of this study show that the phylogenetic position of Glyptothorax fishes is in the interior of the glyptosternoid fishes, which is consistent with the results of He (1996) and Guo et al. (2005, 2007).

## Data Availability

The genome sequence data that support the findings of this study are openly available in GenBank of NCBI at [https://www.ncbi.nlm.nih.gov] (https://www.ncbi.nlm.nih.gov/) under the accession MN396887.
